# The Potential of JAG Ligands as Therapeutic Targets and Predictive Biomarkers in Multiple Myeloma

**DOI:** 10.3390/ijms241914558

**Published:** 2023-09-26

**Authors:** Natalia Platonova, Elisa Lazzari, Michela Colombo, Monica Falleni, Delfina Tosi, Domenica Giannandrea, Valentina Citro, Lavinia Casati, Domenica Ronchetti, Niccolò Bolli, Antonino Neri, Federica Torricelli, Leslie A. Crews, Catriona H. M. Jamieson, Raffaella Chiaramonte

**Affiliations:** 1 Department of Health Sciences, Università degli Studi di Milano, 20142 Milan, Italy; natalia.platonova@unimi.it (N.P.); elazzari88@gmail.com (E.L.); michela.colombo@fht.org (M.C.); monica.falleni@unimi.it (M.F.); delfina.tosi@unimi.it (D.T.); domenica.giannandrea@unimi.it (D.G.); valentina.citro@unimi.it (V.C.); lavinia.casati@unimi.it (L.C.); 2 Division of Regenerative Medicine, Department of Medicine, Moores Cancer Center, University of California, La Jolla, CA 92093, USA; lcrews@health.ucsd.edu (L.A.C.); cjamieson@ucsd.edu (C.H.M.J.); 3 UC San Diego Sanford, Stem Cell Institute, La Jolla, CA 92037, USA; 4 Unit of Pathology A.O. San Paolo, Via A. Di Rudinì 8, 20142 Milan, Italy; 5 Department of Oncology and Hemato-Oncology, Università degli Studi di Milano, 20122 Milan, Italy; domenica.ronchetti@unimi.it (D.R.); niccolo.bolli@unimi.it (N.B.); 6 Hematology, Fondazione Cà Granda IRCCS Policlinico, 20122 Milan, Italy; 7 Scientific Directorate, Azienda USL-IRCCS di Reggio Emilia, 42123 Reggio Emilia, Italy; antonino.neri@ausl.re.it; 8 Laboratory of Translational Research, Azienda USL-IRCCS di Reggio Emilia, 42123 Reggio Emilia, Italy; federica.torricelli@ausl.re.it

**Keywords:** JAG1, JAG2, multiple myeloma, proliferation, NOTCH signaling, mouse model, microenvironment

## Abstract

The NOTCH ligands JAG1 and JAG2 have been correlated in vitro with multiple myeloma (MM) cell proliferation, drug resistance, self-renewal and a pathological crosstalk with the tumor microenvironment resulting in angiogenesis and osteoclastogenesis. These findings suggest that a therapeutic approach targeting JAG ligands might be helpful for the care of MM patients and lead us to explore the role of JAG1 and JAG2 in a MM in vivo model and primary patient samples. JAG1 and JAG2 protein expression represents a common feature in MM cell lines; therefore, we assessed their function through JAG1/2 conditional silencing in a MM xenograft model. We observed that JAG1 and JAG2 showed potential as therapeutic targets in MM, as their silencing resulted in a reduction in the tumor burden. Moreover, JAG1 and JAG2 protein expression in MM patients was positively correlated with the presence of MM cells in patients’ bone marrow biopsies. Finally, taking advantage of the Multiple Myeloma Research Foundation (MMRF) CoMMpass global dataset, we showed that JAG2 gene expression level was a predictive biomarker associated with patients’ overall survival and progression-free survival, independently from other main molecular or clinical features. Overall, these results strengthened the rationale for the development of a JAG1/2-tailored approach and the use of JAG2 as a predictive biomarker in MM.

## 1. Introduction

Multiple myeloma (MM) is an incurable plasma cell malignancy with a 5-year survival rate of 52% [1]. Malignant plasma cells mainly reside in the bone marrow (BM). The interplay between MM cells and the surrounding BM tumor microenvironment and the cytokine milieu are vital for MM pathogenesis and its metastatic spread to distant bone sites [2,3,4]. The key role of this pathological interplay captured our interest when it came to exploring the potential contribution of NOTCH signaling, a pivotal pathway involved in sending and receiving signals during cellular communication during both embryonic and adult tissue development, as well as in cancer [5,6].

The activation of NOTCH signaling occurs through the interaction between receptors and ligands situated on the cell membrane. There are four distinct isoforms of NOTCH receptors, NOTCH 1 through 4. The NOTCH ligands are categorized into two families: the Delta family, which comprise DLL1, DLL3 and DLL4, and the Serrate family, which includes JAG1 and JAG2 [7].

MM cells have been documented to exhibit elevated levels of NOTCH receptors and JAG ligands [8]. Notably, analyses of bone marrow biopsies (BMBs) taken from MM patients reveal that CD138+ myeloma cells showcase significant expression of NOTCH1, NOTCH2 and JAG1 proteins when compared to non-malignant BM cells or plasma cells from healthy donors [9]. Among MM patients, those harboring the translocation and cyclin D TC5 group, characterized by t(14;16)(q32;q23) and t(14;20)(q32;q11) translocation [10,11], have a particularly unfavorable prognosis. These translocations involve the transcription factors c-MAF and MAFB, which subsequently activate NOTCH2 gene transcription [10]. The expression of NOTCH1 and JAG1 is found to be upregulated during the progression from the benign phase of monoclonal gammopathy of undetermined significance (MGUS) to MM [12,13]. JAG2 deregulation occurs during the onset of MGUS, driven by multiple mechanisms, including promoter hypomethylation [14] and increased expression of skeletrophin, an ubiquitin ligase necessary for JAG2 activity [15]. In addition, loss of the SMRT/NCoR2 corepressor results in JAG2 promoter acetylation and subsequent increased transcription [16]. Moreover, our group reported NOTCH signaling upregulation in a cellular model mimicking MM cell progression. Specifically, the overexpression of NOTCH family members through modulation of IL-6 signaling in vitro results in the acquisition of aggressive tumor cell features, such as a higher proliferation rate and independence from IL-6 [13].

The overexpression of NOTCH receptors and ligands in MM cells leads to the activation of NOTCH both within tumor cells through homotypic interaction and in the surrounding microenvironment via heterotypic interactions. The consequences of NOTCH signaling activation induce important biological outcomes, such as MM cell proliferation [13,17], development of drug resistance [18,19,20], cell migration [17], tumor angiogenesis [21,22,23], osteoclastogenesis [23,24,25] and inhibition of bone deposition [24,26]. Furthermore, NOTCH signaling activation fosters the establishment of a cytokine milieu that supports tumor growth, which is characterized by immunosuppressive and proinflammatory features [27].

In this study, we focused on the role of JAG ligands in MM cell biology and their clinical potential. Previous in vitro and ex vivo studies demonstrated that the activation of NOTCH signaling induced by JAG ligand overexpression in MM cells promoted cell proliferation [13,14] and self-renewal [28]. Here, we demonstrate that JAG1 and JAG2: (1) exhibit widespread expression in both MM cell lines and MM cells derived from primary BMBs; (2) represent prognostic factors for MM patients, and (3) are tractable as promising therapeutic targets, as demonstrated by a MM xenograft model wherein conditional knockdown (KD) of JAG1 and JAG2 leads to notable effects.

## 2. Results

### 2.1. Expression of JAG1 and JAG2 In Vitro and Their Impact on a MM Xenograft Mouse Model

**Figure 1:**
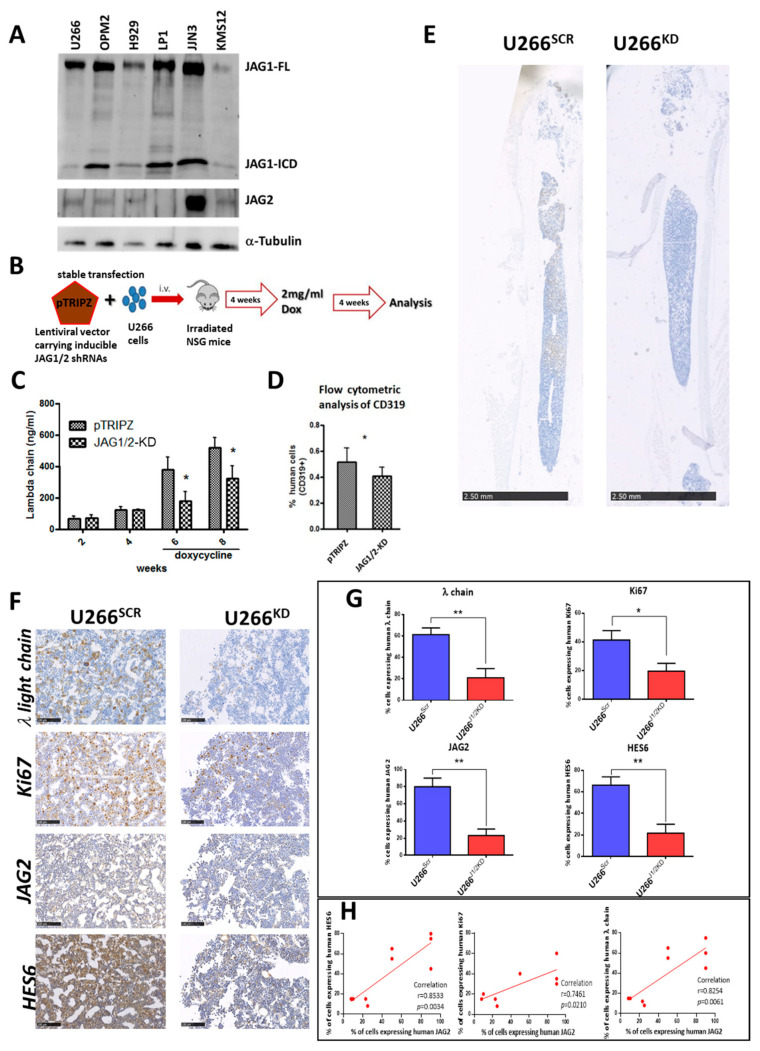
(**A**) Western blot analysis of JAG1 and JAG2 in a panel of 6 MM cell lines (30 µg each). The bands of JAG1 full length (JAG1-FL) and of JAG1-ICD as well as of JAG2 have been detected. α-tubulin was used to normalize the protein extracts loading. The image is representative of two different experiments with similar results. (**B**) Xenograft models of advanced MM were generated as reported in the scheme injecting U266^SCR^ or U266^KD^ cells in NSG (*n* = 4/5 mice/group). Tumor engraftment was measured via (**C**) ELISA for λ Ig human chain in mice serum or (**D**) via flow cytometry analysis of hCD319+ MM cells in the flushed BM cells. Mean values ± SD are shown. Statistical analyses were performed via a two-tailed *t*-test (* = *p* < 0.05). (**E**) Representative immunohistochemistry analyses of mouse femurs show the different growth of U266^SCR^ and U266^KD^ cells (human λ Ig chain in brown) after doxycycline treatment (scale bar 2.50 mm). (**F**) Representative images of immunohistochemical analysis of human λ Ig light chain, Ki67, JAG2 and HES6 (scale bar 100 µm). Photographs were acquired using Nano-Zoomer 2.0. (**G**) Graphs showing the percentage of human λ Ig light chain, Ki67, JAG2 and HES6-positive cells in the femurs of mice bearing U266^SCR^ or U266^KD^ cells. Statistical analyses were performed via unpaired *t*-test; * = *p* < 0.05, ** = *p* < 0.01. (**H**) Correlation analysis between the percentage of positive cells for human JAG2 and those positive for human λ Ig light chain, Ki67 or HES6. Pearson’s correlation coefficient (r) and the *p*-value (calculated using a two tailed *t*-tests) are reported for the correlation analyses.

First, to verify whether JAG1 and JAG2 expression was a common feature of MM cells, we conducted Western blot analyses on a panel of six distinct human cell lines: U266, OPM2, H929, LP1, JJN3 and KMS12. Figure 1A demonstrates varying levels of JAG ligand expression within the examined MM cell lines. Notably, JAG1 exhibited higher expression levels compared to JAG2, with all cell lines expressing at least one of these ligands. Furthermore, we detected the presence of the JAG1 intracellular domain (JAG1-ICD), released subsequent to Notch engagement by ADAM-17 and γ-Secretase [29], across all cell lines.

Given that in vitro analyses of the role of JAG1 and JAG2 in MM [13,21,28] have yet to be corroborated by in vivo evidence, we developed a JAG1/2-modulated MM xenograft murine model using established techniques [30]. This enabled us to compare the growth of a MM cell line after conditional silencing of JAG1 and JAG2. The U266 cell line was chosen for this purpose due to its moderate levels of JAG1 and JAG2 expression. We performed a conditional KD in U266 cells using a pTRIPZ lentiviral vector stably expressing short hairpin RNAs (shRNA) for JAG1 and JAG2 (U266^KD^) or the corresponding scrambled (SCR) shRNAs (U266^SCR^), as previously described [21]. Subsequently, intramedullary MM xenograft models were generated (Figure 1B) via intravenous (i.v.) injection of U266^SCR^ or U266^KD^ cells in previously irradiated NSG mice (*n* = 4/5 mice/group). Tumor engraftment was monitored via ELISA, measuring human λ Ig chain levels in mouse serum. Upon BM engraftment of the transplanted MM cells (4 weeks post i.v. injection), similar λ Ig chain levels were observed in both groups. Then, doxycycline was administered to the mice through their drinking water to induce JAG1 and JAG2 silencing. After a total of 8 weeks post-transplant, the mice were humanely sacrificed and analysis of tumor burden within the two experimental groups was conducted. Human λ Ig chain serum levels determined via ELISA (Figure 1C), percentage of hCD319^+^ MM cells determined via flow cytometry analysis (Figure 1D), and the percentage of MM cells positive for the human λ Ig light chain from murine BM samples were assessed (Figure 1E and Supplementary Table S1). Additionally, the proliferation marker Ki67 and the efficacy of JAG1 and JAG2 silencing were assessed via immunohistochemistry analysis of human JAG2 and of HES6, a proxy for downstream Notch target activation (Figure 1F). Notably, the assessment of human JAG1 expression was impeded due to the cross-reactivity of commercially available anti-human JAG1 antibodies with murine JAG1.

Overall, these analyses showed a clear decrease in tumor burden in mice injected with U266^KD^ cells after treatment with doxycycline in comparison to mice injected with U266^SCR^ cells.

Additionally, both JAG2 expression and NOTCH pathway activation (by HES6 expression) were reduced in the BM of mice engrafted with U266^KD^ cells compared to the U266^SCR^-engrafted group (Figure 1G). This reduction was associated with a statistically significant decrease in the percentage of human U266 cells and in their cell proliferation (Ki67 levels). The expression levels of human JAG2 in U266 cells isolated from BM samples of xenografted mice showed a statistically significant correlation with the amount of tumor cells, their proliferation index and the levels of NOTCH activation (Figure 1H).

Taken together, these in vivo results indicated that the expression of JAG ligands was crucial for MM growth, and that the conditional knockout of JAG1 and JAG2 ligands significantly reduced the tumor burden in our MM xenograft model.

### 2.2. Correlation between JAG1 and JAG2 Expression and Tumor Cell Presence in MM Patients’ Bone Marrow Biopsies

**Figure 2:**
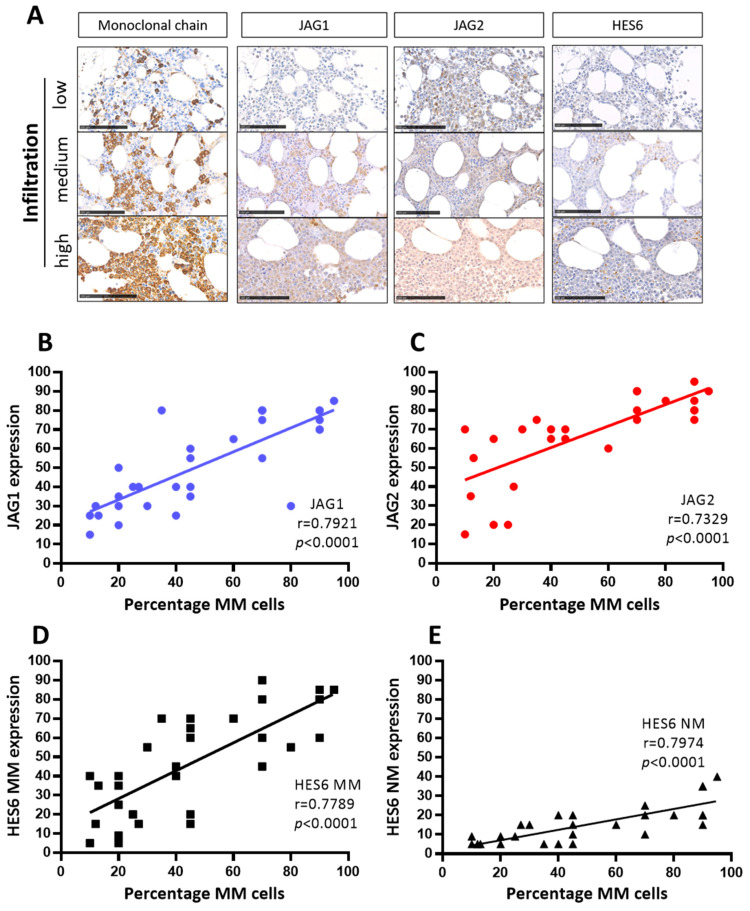
JAG ligands expression and NOTCH pathway activation correlate with the percentage of MM cells in the BM of 34 MM patients at onset. (**A**) Representative images of the antigen immunoreactivity for the monoclonal Ig light chain, JAG1, JAG2, HES6 and Ki67. Photos were acquired via Nano-Zoomer 2.0 and the scale bar is 100 µm. Pearson’s correlation coefficient (r) and the *p*-values are reported for the correlation analyses of the percentage of MM cells positive for monoclonal light chains with, respectively (**B**) JAG1, (**C**) JAG2, (**D**) HES6 in MM cells and (**E**) HES6 in non-myeloma NM cells.

To investigate the potential correlation between the expression of JAG1 and JAG2 and the extent of tumor cell burden in MM patients, we leveraged a set of 34 BMBs obtained from MM patients at disease onset, previously analyzed via immunohistochemistry to identify the presence of monoclonal light chains, JAG ligands, and HES6 [21]. Here, we quantified the percentage of malignant plasma cells (previously classified by three different grades of infiltration) (Supplementary Table S2) and correlated the degree of tumor burden with the expression of JAG ligands and the activation of NOTCH signaling, within MM cells and surrounding BM non-malignant (NM) cells (Figure 2).

Representative images (Figure 2A) and correlation analyses of the percentage of MM cells and JAG1 (Figure 2B), JAG2 (Figure 2C), HES6 in MM cells (Figure 2D), and HES6 in NM non-myeloma cells (Figure 2E) are reported here. The results of these analyses revealed statistically significant positive correlations between the percentage of MM cells in the BMBs, the expression of both JAG1 and JAG2, and the activation of NOTCH signaling in myeloma (HES6 MM) as well as in non-myeloma cells (HES6 NM). These results are consistent with the notion that MM-derived JAG ligands can enhance the growth of MM cells and trigger NOTCH signaling activation in tumor cells and in the surrounding tumor microenvironment.

### 2.3. Clinical Relevance of JAG1 and JAG2 Expression

To gain insight into the clinical relevance of JAG1 and JAG2 expression, we analyzed primary MM patient profiles, from the publicly available Multiple Myeloma Research Foundation (MMRF) CoMMpass database. We stratified a total of 767 MM patients, for whom both RNA-seq data and clinical data were available, in high- versus low-expression groups based on the median cut-off value for either JAG1 or JAG2 expression level across the entire dataset.

**Figure 3:**
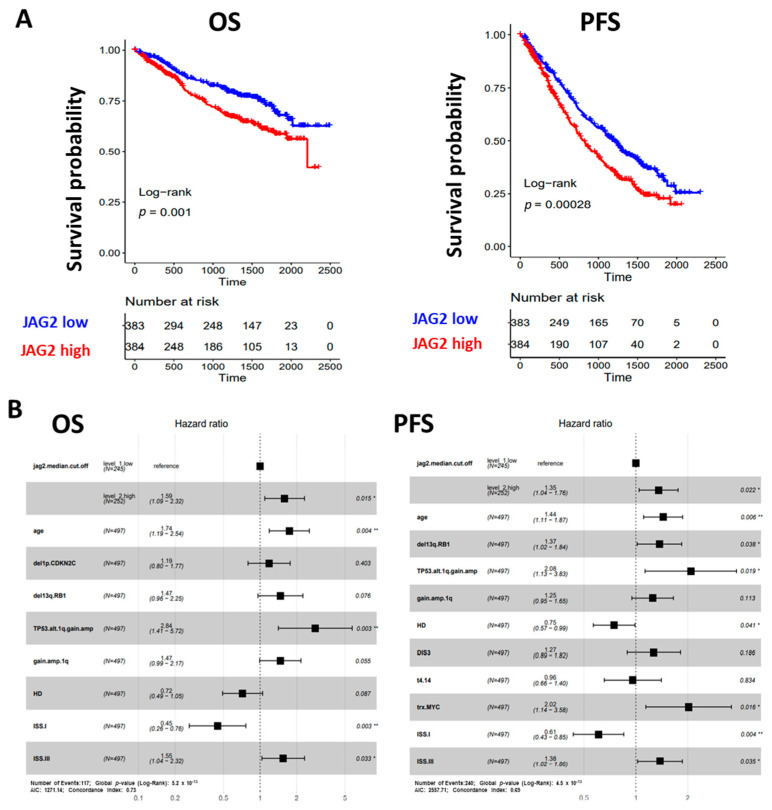
(**A**) Kaplan–Meier survival curves in the CoMMpass global dataset including 767 MM samples: overall survival (OS) on the **left** and progression-free survival (PFS) on the **right**. MM cases were stratified in high and low JAG2 gene expression groups, based on the median expression level across the dataset. Log-rank test *p*-value measuring the global difference between survival curves and number of samples at risk in each group across time are reported. (**B**) Forest plot of cox regression multivariate analysis considering all features with adjusted *p*-value < 0.05 in univariate analysis with regard to OS (**left**) and PFS (**right**) in 497 MM cases. Hazard Ratio, 95% Confidence Interval and Log-rank *p*-value are indicated in the plot for each variable. Significant *p*-value: * ≤ 0.05; ** ≤ 0.01.

Our analysis showed that higher JAG2 expression levels were associated with a poorer clinical outcome in terms of both overall survival (OS) and progression-free survival (PFS), as depicted in Figure 3A. Conversely, while the JAG1 expression level displayed a positive correlation with MM disease progression [13], it did not impact the clinical outcomes of MM patients (Supplementary Figure S1).

To establish whether elevated levels of JAG2 expression could independently predict OS and PFS, we tested the condition of high JAG2 expression and other main molecular or clinical features via Cox regression univariate analysis in a dataset of 497 MM samples for which all information records were available. In terms of OS, a significantly higher risk of death was observed for cases with higher JAG2 expression levels (Hazard Ratio, HR = 1.9, 95% CI 1.3–2.8, BH adj. *p*-value = 0.0024), together with older age (equal or over 65 years), ISS stage III, and distinct molecular variables such as del(1p)/CDKN2C, del(13q)/RB1, and 1q gain/amplification alone or in combination with TP53 alterations; on the contrary, ISS stage I and HD cases showed a 69% and 36% death risk reduction, respectively (Supplementary Figure S2). Moreover, when all significant variables were tested in multivariate analysis for OS, higher JAG2 expression levels retained significance (Figure 3B). Regarding PFS, higher JAG2 expression levels significantly correlated with a higher risk of disease progression (Hazard Ratio, HR = 1.7, 95% CI 1.3–2.2, BH adj. *p*-value = 0.0057), as well as older age, ISS stage III, and distinct molecular variables such as del(13q)/RB1, 1q gain/amplification alone or in combination with TP53 alterations, t(4;14) translocation, MYC translocation, and the presence of DIS3 mutations; conversely, ISS stage I and HD cases showed a 56% and 34% progression risk reduction, respectively (Supplementary Figure S2). Moreover, when all significant variables were tested in multivariate analysis for PFS, higher JAG2 expression levels retained significance (Figure 3B).

Overall, these data from the CoMMpass cohort demonstrated the clinical impact of JAG2 expression levels in MM, independently from known genetic prognostic factors.

## 3. Discussion

The lack of a definitive cure for patients with MM has driven scientific research toward new directions. Several studies have demonstrated that inhibiting the NOTCH pathway effectively counteracts various malignant features of MM, including cell proliferation, survival and self-renewal [13,17,18,28] and the aberrant communication between MM cells and their surrounding BM microenvironment, which promotes drug resistance, tumor angiogenesis and bone disease [18,19,21,22,23,24,26]. Consequently, the NOTCH pathway may be considered a promising therapeutic target [31].

Nevertheless, clinical trials have revealed that pan-NOTCH inhibition with ϒ-Secretase inhibitors induces gastrointestinal toxicity [32] as a result of their impact on goblet cells [33]. Therefore, a more precise approach that targets specific NOTCH signaling members, rather than blocking the entire pathway, should be considered.

From a safety standpoint, inhibiting JAG ligands represents a safer alternative to pan-NOTCH inhibition. Recent evidence highlights that blocking NOTCH pathway activation mediated by either the JAG or DLL family of ligands strongly mitigates side effects [34].

Building on these promising observations, we sought to provide reliable experimental evidence that strengthens the therapeutic potential of JAG ligands. First, we demonstrated the expression of JAG ligands across a panel of six MM cell lines, which represents a general feature of MM. The role of JAG1 and JAG2 expression in vitro has been previously reported by our group and others. JAG ligands trigger the activation of NOTCH receptors via both homotypic and heterotypic interactions [18], thereby inducing NOTCH signaling activation not only in within MM cells, but also in the tumor microenvironment, causing the pro-tumor effects associated with NOTCH activation [13,14,18]. Additionally, JAG2 expression levels correlate with increased self-renewal of MM cells [28]. In this manner, we aimed to bolster the evidence supporting the role of JAG ligands in regulating tumor growth in an in vivo setting. For this purpose, we took advantage of an immunodeficient murine model (NSG mouse) xenografted with the U266 MM cell line, which carried an inducible vector for JAG1 and JAG2 KD. Our previous research indicated that NOTCH signaling influences the localization of MM cells within the bone marrow [17]. To specifically evaluate the effect of dual JAG ligand KD exclusively on tumor burden, U266 cells transduced with a lentiviral vector expressing doxycycline-inducible shRNAs for JAG1 and JAG2 were injected in NGS mice, and JAG1 and JAG2 silencing was induced after MM cell engraftment. Immunohistochemistry analyses demonstrated that the conditional knockdown of JAG ligands significantly impaired NOTCH signaling activation and negatively regulated MM cell proliferation. Most importantly, at 2 and 4 weeks after doxycycline administration, there was a significant reduction in tumor burden. Collectively, these in vivo findings provide compelling confirmation that JAG1 and JAG2 hold substantial therapeutic promise for MM treatment by impacting MM cell proliferation.

We validated the results of our in vivo experiments by examining BMBs from MM patients. In doing so, we showed that the expression levels of JAG ligands correlated with the activation of NOTCH signaling in both MM cells as well as in the neighboring non-myeloma cells. Most importantly, we established that the greater the expression of JAG ligands, the higher the number of tumor cells detected in the BMBs. This finding confirmed previous in vitro evidence [18] as well as our in vivo results, collectively suggesting that the expression of JAG ligands is associated with MM cell growth.

The potential of JAG ligands as therapeutic targets received further validation through the examination of their expression in MM patient tumor cells. Our analysis, involving a correlation between JAG1 or JAG2 gene expression and clinical data from 767 MM patients (CoMMpass database), unveiled a positive association between JAG2 expression levels and an increased risk of death and disease progression, independently from other known genetic prognostic factors such as older age, ISS stage III, and distinct molecular variables such as del(1p)/CDKN2C, del(13q)/RB1, 1q gain/amplification alone or in combination with TP53 alterations, t(4;14) translocation, MYC translocation, and the presence of DIS3 mutations. By contrast, the analysis on the CoMMpass database did not reveal any association between JAG1 expression levels and OS and PFS. However, a previous gene expression profiling study conducted on a proprietary dataset of 129 MM cases indicated that JAG1 was overexpressed in MM compared to normal controls, with the highest expression levels observed in primary plasma cell leukemia [13]. In conclusion, our findings advocate for JAG2 as a predictive marker for adverse prognosis in MM, and both JAG ligands emerge as potential targets for anti-myeloma therapy.

Interestingly, the therapeutic potential of JAG ligands has already been explored in different tumor settings [35,36,37,38], specifically in breast cancer with brain and bone metastasis [35,38]. Similarly to our results, treatment with a JAG1-neutralizing antibody in vivo strongly inhibited brain metastasis growth in triple-negative breast cancer and sensitizes bone metastasis to chemotherapy, without evident toxicity [35,38]. Our group and others have proposed two distinct strategies involving small molecules [39] or neutralizing antibodies [35,38] to disrupt the JAG-NOTCH interaction. In summary, we believe that our study contributes substantial evidence to support the rationale for developing targeted therapeutic strategies focused on JAG in MM treatment.

## 4. Materials and Methods

### 4.1. Cell Lines

MM cell lines, OPM2 (ACC-50) LP1 (ACC-41), KMS12 (ACC-551) and JJN3 (ACC-541) were purchased from the DSMZ collection of microorganisms; U266 (ATCC^®^ TIB-196) and H929 (ATCC^®^ CRL-906) were purchased from the American Type Culture Collection. Cells were cultured in RPMI1640 medium (Euroclone, Pero, Italy) supplemented with 10% fetal bovine serum (Euroclone, Italy), 100 U/mL penicillin/streptomycin (Microgem, Napoli, Italy) and 2 mM l-glutamine (Microgem, Italy).

### 4.2. Western Blot

Western blot analysis of cellular extracts was performed as described previously [40]. Briefly, protein cell extracts were lysed in RIPA lysis buffer containing a protease inhibitor cocktail (Sigma Aldrich, Milano, Italy). Protein samples (30 μg) were loaded on PAGE-SDS gel electrophoresis and transferred onto a nitrocellulose membrane (Hybond-ECL, Amersham Bioscience, Milano, Italy), followed by blocking with 5% nonfat milk in TBS-T (20 mM Tris-Cl, pH 7.5, 150 mM NaCl, 0.05% Tween-20). Membranes were incubated overnight at 4 °C with α-tubulin antibody (sc-12462, Santa Cruz Biotechnology, Dallas, TX, USA), JAG1 antibody (n.70109, Cell Signaling Technology, Danvers, MA, USA) and JAG2 antibody (n.2205, Cell Signaling Technology) followed by incubation with the appropriated HRP-conjugated species-specific secondary antibody (Promega, Milano, Italy). Chemiluminescence was detected using the Western Bright ECL HRP substrate (Advansta Inc., San Jose, CA, USA). Chemiluminescent signal was captured using the ChemiDoc Imaging System (Bio-Rad Laboratories, Inc., Hercules, CA, USA).

### 4.3. Murine Model

All mouse studies were completed in accordance with the University of California San Diego Animal Care Program regulations, with approval from the Institutional Animal Care and Use Committee. Immunocompromised NSG mice were obtained commercially from Jackson Laboratories and maintained and treated in animal facilities at UC San Diego according to IACUC-approved protocols.

JAG1 and JAG2 conditional KD was performed on U266 cells as described previously [21]. After selection with puromycin, the U266^SCR^ and U266^KD^ cells (10^7^ cells in 200 μL PBS) were intravenously injected into adult NSG mice previously irradiated at 210cGy (*n* = 4/5 mice per group). Tumor engraftment was monitored every two weeks post-transplant by measuring the levels of human λ Ig chain via ELISA in mice serum. Doxycycline was administered in drinking water ad libitum (2 mg/mL) starting at week 5 to induce JAG1/2 silencing. After 4 weeks, the mice were sacrificed.

Femurs isolated from treated animals were formalin-fixed, decalcified, and routinely embedded in paraffine, as described for human BM samples. Serial 3-micron sections containing bone marrow cells were analyzed as previously described [21]. Immunoreactivity for λ Ig light chain, Ki67, JAG2, and HES6 was compared in animals engrafted with U266^SCR^ and U266^KD^ cells.

### 4.4. Serum Immunoglobulin Light Chain Level Assessment

Peripheral blood was collected from each mouse via retro-orbital bleeding, while animals were anesthetized with isoflurane. Next, 10–50 μL of serum were analyzed via an enzyme-linked immunosorbent assay as described before [41] using kits for Human lambda or kappa chains (Bethyl Laboratories Inc., Montgomery, TX, USA) according to the manufacturer’s guidelines.

### 4.5. Flow Cytometry Analysis

BM single-cell suspensions were derived from MM-engrafted animals by flushing femurs with sterile PBS solution. Cells were stained with LIVE/DEAD fixable near-IR viability dye (Thermofisher, Waltham, MA, USA) at 1:1000 in PBS. Unbound viability dye was then washed out with PBS and non-specific Fc receptor-mediated antibody binding was blocked via incubation with mouse and human FcR blocking reagent (BD Biosciences, Franklin Lakes, NJ, USA). Samples were then stained with anti-human CD319-PE (Miltenyi Biotec, San Diego, CA, USA) at 4 °C in the dark and fixed with 1% PFA for 10 min at 4 °C. Samples were then acquired by MACSQuant (Miltenyi, Biotec, San Diego, CA, USA).

### 4.6. Multi-Omics Data in CoMMpass Study

Multi-omics data about bone marrow MM samples at baseline (BM_1) were publicly accessible from the MMRF CoMMpass Study (https://research.themmrf.org/ (accessed on 16 October 2020), which included more than 1000 MM patient samples collected from several sites worldwide and retrieved from the Interim Analysis 15a (MMRF_CoMMpass_IA15a, accessed on 16 October 2020). Transcript per Million (TPM) reads values for JAG1 and JAG2 transcripts were retrieved using Salmon gene expression quantification data (MMRF_CoMMpass_IA15a_E74GTF_Salmon_V7.2_Filtered_Gene_TPM) in 774 BM_1 MM patients.

Overall survival (OS) and progression-free survival (PFS) clinical data were analyzed for 767 MM patients for which both RNA-seq expression and survival information were available. Non-synonymous (NS) somatic mutation variants and counts data were obtained from whole-exome sequencing (WES) analyses, main IgH translocations were inferred from RNA-seq spike expression estimates of known target genes, and Copy Number Alteration (CNA) data were retrieved by means of Next-generation Sequencing (NGS)-based fluorescence in situ hybridization (FISH) [42] in 497 MM cases for which all data were available [43]. The presence of a specific CNA was considered when it occurred in at least one of the investigated cytoband at a 20% cut-off for each considered chromosomal aberration, as previously reported [43].

### 4.7. Survival Analysis

Survival analyses were performed using survival and survminer packages in R Bioconductor (version 4.1.2). The median cut-point value was used to stratify MM cases of CoMMpass cohort in high- and low-JAG1 or JAG2 expression groups.

### 4.8. Statistical Analysis

Linear correlation analysis between value sets was performed using the GraphPad software (version 6.1) to compute the Pearson correlation coefficients (r); *p*-values were calculated via a two-tailed *t*-test with a 95% confidence interval.

## Data Availability

The data generated during this study are included in this published article. The analyzed MMRF CoMMpass data are available at https://research.themmrf.org/ (accessed on 16 October 2020) and retrieved from the Interim Analysis 15a (MMRF_CoMMpass_IA15a, 16 October 2020).

## Supplementary Materials

The supporting information can be downloaded at: https://www.mdpi.com/article/10.3390/ijms241914558/s1.

## References

[1] Guzdar A., Costello C. (2020). Supportive Care in Multiple Myeloma. Curr. Hematol. Malig. Rep..

[2] Jurisic V. (2020). Multiomic analysis of cytokines in immuno-oncology. Expert Rev. Proteom..

[3] Konjević G.M., Vuletić A.M., Mirjačić Martinović K.M., Larsen A.K., Jurišić V.B. (2019). The role of cytokines in the regulation of NK cells in the tumor environment. Cytokine.

[4] Moschetta M., Kawano Y., Sacco A., Belotti A., Ribolla R., Chiarini M., Giustini V., Bertoli D., Sottini A., Valotti M. (2017). Bone Marrow Stroma and Vascular Contributions to Myeloma Bone Homing. Curr. Osteoporos. Rep..

[5] Fiuza U.M., Arias A.M. (2007). Cell and molecular biology of Notch. J. Endocrinol..

[6] Kushwah R., Guezguez B., Lee J.B., Hopkins C.I., Bhatia M. (2014). Pleiotropic roles of Notch signaling in normal, malignant, and developmental hematopoiesis in the human. EMBO Rep..

[7] Aster J.C., Pear W.S., Blacklow S.C. (2017). The Varied Roles of Notch in Cancer. Annu. Rev. Pathol..

[8] Milner L.A. (2004). Notch signaling: A key to the pathogenesis of multiple myeloma?. Blood.

[9] Jundt F., Pröbsting K.S., Anagnostopoulos I., Muehlinghaus G., Chatterjee M., Mathas S., Bargou R.C., Manz R., Stein H., Dörken B. (2004). Jagged1-induced Notch signaling drives proliferation of multiple myeloma cells. Blood.

[10] Johnson S.K., Heuck C.J., Albino A.P., Qu P., Zhang Q., Barlogie B., Shaughnessy J.D. (2011). The use of molecular-based risk stratification and pharmacogenomics for outcome prediction and personalized therapeutic management of multiple myeloma. Int. J. Hematol..

[11] Hideshima T., Bergsagel P.L., Kuehl W.M., Anderson K.C. (2004). Advances in biology of multiple myeloma: Clinical applications. Blood.

[12] Škrtić A., Korać P., Krišto D.R., Stojisavljević R.A., Ivanković D., Dominis M. (2010). Immunohistochemical analysis of NOTCH1 and JAGGED1 expression in multiple myeloma and monoclonal gammopathy of undetermined significance. Hum. Pathol..

[13] Colombo M., Galletti S., Bulfamante G., Falleni M., Tosi D., Todoerti K., Lazzari E., Crews L.A., Jamieson C.H., Ravaioli S. (2016). Multiple myeloma-derived Jagged ligands increases autocrine and paracrine interleukin-6 expression in bone marrow niche. Oncotarget.

[14] Houde C., Li Y., Song L., Barton K., Zhang Q., Godwin J., Nand S., Toor A., Alkan S., Smadja N.V. (2004). Overexpression of the NOTCH ligand JAG2 in malignant plasma cells from multiple myeloma patients and cell lines. Blood.

[15] Takeuchi T., Adachi Y., Ohtsuki Y. (2005). Skeletrophin, a novel ubiquitin ligase to the intracellular region of Jagged-2, is aberrantly expressed in multiple myeloma. Am. J. Pathol..

[16] Ghoshal P., Nganga A.J., Moran-Giuati J., Szafranek A., Johnson T.R., Bigelow A.J., Houde C.M., Avet-Loiseau H., Smiraglia D.J., Ersing N. (2009). Loss of the SMRT/NCoR2 corepressor correlates with JAG2 overexpression in multiple myeloma. Cancer Res..

[17] Mirandola L., Apicella L., Colombo M., Yu Y., Berta D.G., Platonova N., Lazzari E., Lancellotti M., Bulfamante G., Cobos E. (2013). Anti-Notch treatment prevents multiple myeloma cells localization to the bone marrow via the chemokine system CXCR4/SDF-1. Leukemia.

[18] Colombo M., Garavelli S., Mazzola M., Platonova N., Giannandrea D., Colella R., Apicella L., Lancellotti M., Lesma E., Ancona S. (2020). Multiple myeloma exploits Jagged1 and Jagged2 to promote intrinsic and bone marrow-dependent drug resistance. Haematologica.

[19] Muguruma Y., Yahata T., Warita T., Hozumi K., Nakamura Y., Suzuki R., Ito M., Ando K. (2017). Jagged1-induced Notch activation contributes to the acquisition of bortezomib resistance in myeloma cells. Blood Cancer J..

[20] Nefedova Y., Cheng P., Alsina M., Dalton W.S., Gabrilovich D.I. (2004). Involvement of Notch-1 signaling in bone marrow stroma-mediated de novo drug resistance of myeloma and other malignant lymphoid cell lines. Blood.

[21] Palano M.T., Giannandrea D., Platonova N., Gaudenzi G., Falleni M., Tosi D., Lesma E., Citro V., Colombo M., Saltarella I. (2020). Jagged Ligands Enhance the Pro-Angiogenic Activity of Multiple Myeloma Cells. Cancers.

[22] Saltarella I., Frassanito M.A., Lamanuzzi A., Brevi A., Leone P., Desantis V., Di Marzo L., Bellone M., Derudas D., Ribatti D. (2019). Homotypic and Heterotypic Activation of the Notch Pathway in Multiple Myeloma-Enhanced Angiogenesis: A Novel Therapeutic Target?. Neoplasia.

[23] Giannandrea D., Platonova N., Colombo M., Mazzola M., Citro V., Adami R., Maltoni F., Ancona S., Dolo V., Giusti I. (2022). Extracellular vesicles mediate the communication between multiple myeloma and bone marrow microenvironment in a NOTCH dependent way. Haematologica.

[24] Sabol H.M., Amorim T., Ashby C., Halladay D., Anderson J., Cregor M., Sweet M., Nookaew I., Kurihara N., Roodman G.D. (2022). Notch3 signaling between myeloma cells and osteocytes in the tumor niche promotes tumor growth and bone destruction. Neoplasia.

[25] Sekine C., Koyanagi A., Koyama N., Hozumi K., Chiba S., Yagita H. (2012). Differential regulation of osteoclastogenesis by Notch2/Delta-like 1 and Notch1/Jagged1 axes. Arthritis Res. Ther..

[26] Delgado-Calle J., Anderson J., Cregor M.D., Hiasa M., Chirgwin J.M., Carlesso N., Yoneda T., Mohammad K.S., Plotkin L.I., Roodman G.D. (2016). Bidirectional Notch Signaling and Osteocyte-Derived Factors in the Bone Marrow Microenvironment Promote Tumor Cell Proliferation and Bone Destruction in Multiple Myeloma. Cancer Res..

[27] Sabol H.M., Delgado-Calle J. (2021). The multifunctional role of Notch signaling in multiple myeloma. J. Cancer Metastasis Treat..

[28] Chiron D., Maïga S., Descamps G., Moreau P., Le Gouill S., Marionneau S., Ouiller T., Moreaux J., Klein B., Bataille R. (2012). Critical role of the NOTCH ligand JAG2 in self-renewal of myeloma cells. Blood Cells Mol. Dis..

[29] Pelullo M., Quaranta R., Talora C., Checquolo S., Cialfi S., Felli M.P., Te Kronnie G., Borga C., Besharat Z.M., Palermo R. (2014). Notch3/Jagged1 circuitry reinforces notch signaling and sustains T-ALL. Neoplasia.

[30] Lazzari E., Mondala P.K., Santos N.D., Miller A.C., Pineda G., Jiang Q., Leu H., Ali S.A., Ganesan A.-P., Wu C.N. (2017). Alu-dependent RNA editing of GLI1 promotes malignant regeneration in multiple myeloma. Nat. Commun..

[31] Briot A., Iruela-Arispe M.L. (2015). Blockade of specific NOTCH ligands: A new promising approach in cancer therapy. Cancer Discov..

[32] Wong G.T., Manfra D., Poulet F.M., Zhang Q., Josien H., Bara T., Engstrom L., Pinzon-Ortiz M., Fine J.S., Lee H.J.J. (2004). Chronic treatment with the gamma-secretase inhibitor LY-411,575 inhibits beta-amyloid peptide production and alters lymphopoiesis and intestinal cell differentiation. J. Biol. Chem..

[33] Milano J., McKay J., Dagenais C., Foster-Brown L., Pognan F., Gadient R., Jacobs R.T., Zacco A., Greenberg B., Ciaccio P.J. (2004). Modulation of notch processing by gamma-secretase inhibitors causes intestinal goblet cell metaplasia and induction of genes known to specify gut secretory lineage differentiation. Toxicol. Sci. Off. J. Soc. Toxicol..

[34] Kangsamaksin T., Murtomaki A., Kofler N.M., Cuervo H., Chaudhri R.A., Tattersall I.W., Rosenstiel P.E., Shawber C.J., Kitajewski J. (2015). NOTCH decoys that selectively block DLL/NOTCH or JAG/NOTCH disrupt angiogenesis by unique mechanisms to inhibit tumor growth. Cancer Discov..

[35] Zheng H., Bae Y., Kasimir-Bauer S., Tang R., Chen J., Ren G., Yuan M., Esposito M., Li W., Wei Y. (2017). Therapeutic Antibody Targeting Tumor- and Osteoblastic Niche-Derived Jagged1 Sensitizes Bone Metastasis to Chemotherapy. Cancer Cell.

[36] Sierra R.A., Trillo-Tinoco J., Mohamed E., Yu L., Achyut B.R., Arbab A., Bradford J.W., Osborne B.A., Miele L., Rodriguez P.C. (2017). Anti-Jagged Immunotherapy Inhibits MDSCs and Overcomes Tumor-Induced Tolerance. Cancer Res..

[37] Steg A.D., Katre A.A., Goodman B., Han H.-D., Nick A.M., Stone R.L., Coleman R.L., Alvarez R.D., Lopez-Berestein G., Sood A.K. (2011). Targeting the notch ligand JAGGED1 in both tumor cells and stroma in ovarian cancer. Clin. Cancer Res. Off. J. Am. Assoc. Cancer Res..

[38] Masiero M., Li D., Whiteman P., Bentley C., Greig J., Hassanali T., Watts S., Stribbling S., Yates J., Bealing E. (2019). Development of Therapeutic Anti-JAGGED1 Antibodies for Cancer Therapy. Mol. Cancer Ther..

[39] Platonova N., Parravicini C., Sensi C., Paoli A., Colombo M., Neri A., Eberini I., Chiaramonte R. (2017). Identification of small molecules uncoupling the Notch::Jagged interaction through an integrated high-throughput screening. PLoS ONE.

[40] Platonova N., Manzo T., Mirandola L., Colombo M., Calzavara E., Vigolo E., Cermisoni G.C., De Simone D., Garavelli S., Cecchinato V. (2015). PI3K/AKT signaling inhibits NOTCH1 lysosome-mediated degradation. Genes Chromosomes Cancer.

[41] Jurisic V., Colovic M. (2002). Correlation of sera TNF-alpha with percentage of bone marrow plasma cells, LDH, beta2-microglobulin, and clinical stage in multiple myeloma. Med. Oncol..

[42] Miller C., Yesil J., Derome M., Donnelly A., Marrian J., McBride K., Network M.C., Auclair D., Keats J.J. (2016). A Comparison of Clinical FISH and Sequencing Based FISH Estimates in Multiple Myeloma: An Mmrf Commpass Analysis. Blood.

[43] Todoerti K., Ronchetti D., Favasuli V., Maura F., Morabito F., Bolli N., Taiana E., Neri A. (2022). DIS3 mutations in multiple myeloma impact the transcriptional signature and clinical outcome. Haematologica.

