# Supplementary material for: The Potential of JAG Ligands as Therapeutic Targets and Predictive Biomarkers in Multiple Myeloma
Source: Int J Mol Sci. 2023 Sep 26;24(19):14558. doi: 10.3390/ijms241914558 (PMC10572399; doi:10.3390/ijms241914558)
Supplement: Supplementary file 1 [file ijms-24-14558-s001.zip › ijms-2556896-supplementary.pdf]

JAG1 expression level

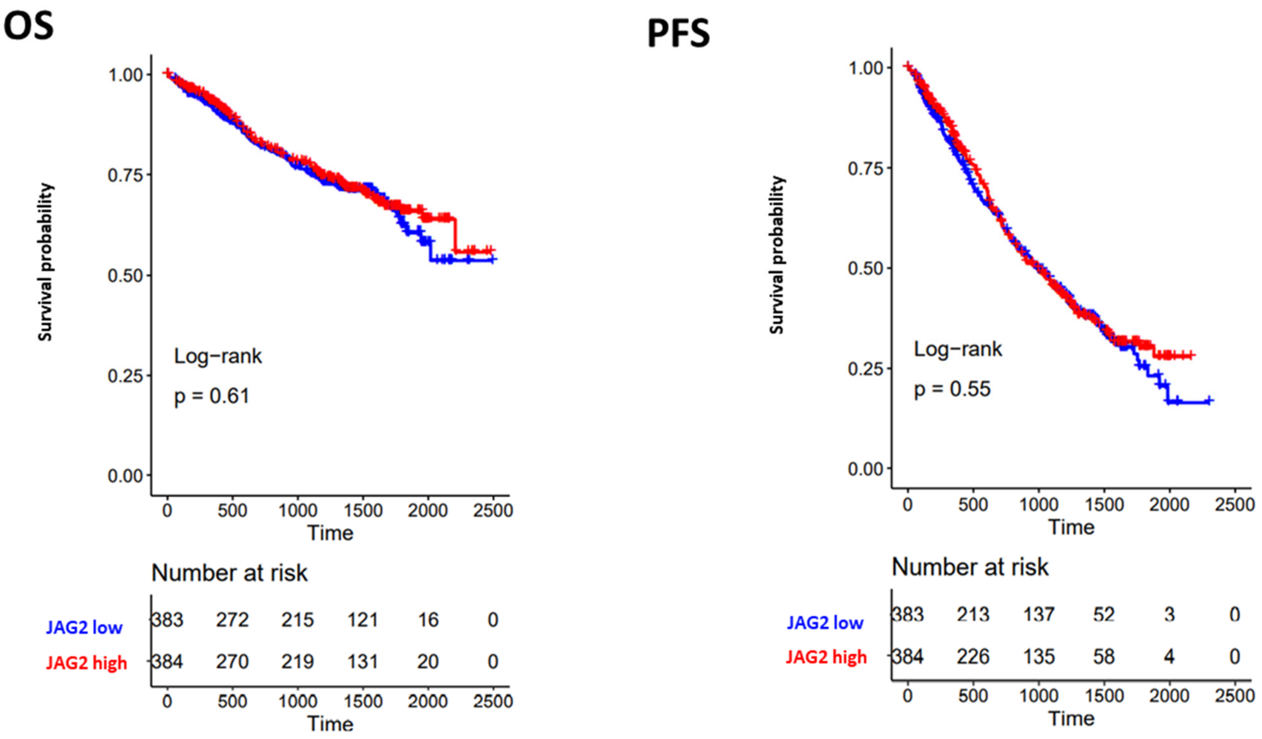

Figure S1: Kaplan-Meier survival curves in 767 MM with JAG1 expression, molecular and clinical data available. Log-rank test p-value measuring the global difference between survival curves and number of samples at risk in each group across time are reported.

A

Variable	N (%)	OS Univariate Cox Analysis		
		HR (95% CI)	P-value	Adj. P-value
high *JAG2*	252(50)	1.9 (1.3-2.8)	0.000709	**0.00248****
Age (≥ 65 yrs)	206 (41)	2.12 (1.46-3.08)	0.0000868	**0.0005*****
ISS I	187 (38)	0.31 (0.19-0.50)	1.86E-06	**0.00002******
ISS II	171 (34)	1.13 (0.78-1.64)	0.524	0.6875
ISS III	139 (28)	2.45 (1.70-3.53)	1.62E-06	**0.00002******
del(1p)/CDKN2C	143 (29)	1.61 (1.10-2.35)	0.0148	**0.03807***
del(13q)/RB1	258 (52)	2.11 (1.44-3.09)	0.000119	**0.00045*****
HD	281 (56)	0.64 (0.44-0.92)	0.0149	**0.0347***
TP53.alterations	40 (8)	1.05 (0.53-2.07)	0.892	0.892
1q21 gain/amp	164 (33)	1.68 (1.17-2.43)	0.0051	**0.0153***
TP53.alterations + 1q21 gain/amp	19 (4)	3.63 (1.89-6.97)	0.000103	**0.0005*****
DIS3 mut	50 (10)	1.63 (1.06-2.50)	0.0264	0.052
N-RAS mut	117 (23)	0.90 (0.62-1.32)	0.596	0.732
K-RAS mut	121 (24)	1.07 (0.77-1.49)	0.694	0.762
BRAF mut	36 (7)	1.18 (0.60-2.31)	0.628	0.732
FAM46C mut	49 (10)	0.78 (0.42-1.43)	0.419	0.586
TRAF3 mut	38 (7)	0.42 (0.17-1.06)	0.0668	0.107
t(11;14)	102 (20)	0.92 (0.58-1.46)	0.726	0.762
t(4;14)	69 (14)	1.63 (1.04-2.55)	0.0335	0.058
MAF.trx	33 (7)	1.96 (1.08-3.57)	0.0276	0.052
MYC.trx	20 (4)	1.92 (0.94-3.94)	0.0748	0.112

B

Variable	N (%)	PFS Univariate Cox Analysis		
		HR (95% CI)	P-value	Adj. P-value
high *JAG2*	252 (50)	1.7 (1.3-2.2)	0.00191	**0.00573****
Age (≥ 65 yrs)	206 (41)	1.59 (1.23-2.05)	0.000372	**0.0019****
ISS I	187 (38)	0.44 (0.34-0.61)	0.00000012	**0.00000*******
ISS II	171 (34)	1.2 (0.91-1.5)	0.213	0.319
ISS III	139 (28)	1.9 (1.48-2.5)	0.00000114	**0.00001******
del(1p)/CDKN2C	143 (29)	1.3 (0.98-1.71)	0.0646	0.104
del(13q)/RB1	258 (52)	1.7 (1.32-2.21)	0.0000471	**0.00032*****
HD	281 (56)	0.66 (0.52-0.86)	0.00166	**0.0057****
TP53.alterations	40 (8)	0.85 (0.52-1.4)	0.511	0.670
1q21 gain/amp	164 (33)	1.52 (1.17-1.97)	0.00151	**0.0057****
TP53.alterations + 1q21 gain/amp	19 (4)	2.46 (1.38-4.41)	0.00243	**0.0063****
DIS3 mut	50 (10)	1.62 (1.18-2.22)	0.00308	**0.0071****
N-RAS mut	117 (23)	1.01 (0.79-1.29)	0.924	0.924
K-RAS mut	121 (24)	1.03 (0.80-1.31)	0.827	0.868
BRAF mut	36 (7)	0.88 (0.52-1.48)	0.626	0.773
FAM46C mut	49 (10)	1.07 (0.74-1.52)	0.728	0.804
TRAF3 mut	38 (7)	0.80 (0.52-1.22)	0.297	0.415
t(11;14)	102 (20)	0.94 (0.68-1.30)	0.694	0.804
t(4;14)	69 (14)	1.6 (1.14-2.23)	0.00612	**0.012***
MAF.trx	33 (7)	1.63 (1.03-2.57)	0.0376	0.065
MYC.trx	20 (4)	1.94 (1.13-3.33)	0.0163	**0.031***

Figure S2: Results of Cox regression univariate analysis using OS **(A)** or PFS **(B)** data on JAG2 expression groups, age equal to or greater than 65 years, ISS subgroups and main molecular alterations in 497 BM-1 MM cases for which all data were available. Number (N) of positive cases is indicated for each variable. Hazard Ratio (HR), 95% Confidence Interval and Log-rank p-value are reported for each variable. In red bold are depicted all significant variables after BH correction. *=p≤0.05; **=p≤0.01;***=p≤0.001;****p≤0.0001

Conditions	λ chain	Ki67	HES6	JAG2
Non transplanted	-	-	-	-
U266^{SCR}	60%	35%	80%	90%
U266^{SCR}	75%	60%	75%	90%
U266^{SCR}	45%	30%	45%	90%
U266^{SCR}	65%	40%	65%	50%
U266$^{J1/2KD}$	55%	40%	55%	50%
U266$^{J1/2KD}$	15%	20%	15%	10%
U266$^{J1/2KD}$	15%	15%	15%	8%
U266$^{J1/2KD}$	8%	8%	8%	25%
U266$^{J1/2KD}$	12%	15%	15%	23%

Table S1. Summary table of the immunohistochemical results on the different bone marrow samples from mice xenografted with U266^{SCR} and U266$^{J1/2KD}$ cells.

Patient N	Light Chain	Infiltrating cells	JAG1	JAG2	Hes6 MM	Hes6 NM
1	κ	20	50	20	5	5
2	λ	12	30	35	15	5
3	λ	20	30	20	9	5
4	λ	25	40	20	20	9
5	λ	10	15	15	5	9
6	λ	20	20	65	35	5
7	κ	10	25	70	40	5
8	κ	20	35	65	40	9
9	λ	20	30	65	25	5
10	λ	13	25	55	35	5
11	λ	40	25	65	40	5
12	κ	30	30	70	55	15
13	λ	45	35	70	15	15
14	κ	45	40	65	20	5
15	κ	40	40	70	45	20
16	κ	27	40	40	15	15
17	κ	45	85	35	50	10
18	κ	45	60	70	60	10
19	κ	35	80	75	70	5
20	λ	45	55	65	65	15
21	κ	45	60	70	70	20
22	κ	80	30	85	55	20
23	κ	90	80	75	60	15
24	λ	60	65	60	70	15
25	λ	70	80	90	80	10
26	κ	90	70	95	85	20
27	κ	70	75	75	45	20
28	κ	70	55	80	60	25
29	κ	90	80	80	80	35
30	κ	70	80	80	N.A.	25
31	κ	90	75	85	80	20
32	λ	95	85	90	85	40
33	κ	90	70	80	80	35
34	κ	70	80	90	90	20

Table S2. Summary table of the immunohistochemical results on the collected bone marrow biopsies (BMB) of 34 MM patients at disease onset analyzed for the immunoreactivity to monoclonal light chains and the percentage of malignant plasma cells, JAG ligands and HES6 expression in MM cells and BM non-malignant (NM) surrounding cells. Data on this table were previously published (15) with the exception of the percentage infiltrating MM cells obtained by a microscopy analysis of previously stained BMBs (15).
